# Can heart failure phenotypes be predicted by cardiac remodelling peripartum or postpartum?

**DOI:** 10.3389/fcvm.2024.1409183

**Published:** 2024-08-06

**Authors:** Megha Agarwal, Paul Leeson, Jamie Kitt

**Affiliations:** Cardiovascular Clinical Research Facility, RDM Division of Cardiovascular Medicine, University of Oxford, Oxford, United Kingdom

**Keywords:** heart failure, hypertensive pregnancy, pre-eclampsia, cardiac remodelling, women

## Abstract

Hypertension during pregnancy affects up to 10% of pregnancies and is associated with significant cardiovascular morbidity and mortality. In the short-term it can result in pre-eclampsia, haemolysis, elevated liver enzymes and low platelets (HELLP) syndrome, or even hypertension associated acute heart failure, all of which may necessitate pre-term delivery to prevent maternal or neonatal death. In the long term, a history of gestational hypertension and pre-eclampsia significantly increases the risk of future cardiovascular disease including chronic hypertension, coronary artery disease, heart failure and stroke. This review explores our current level of knowledge of the phenotypes of heart failure, paying particular attention to those specific to women, and the role of pregnancy and non-pregnancy related risk factors in the development of this condition. We discuss why women with hypertensive pregnancy may be disproportionately affected by heart failure with preserved ejection fraction (HFpEF) and whether a unique phenotype of heart failure unique to hypertensive pregnancy exists. Finally, we explore how future cardiovascular risk may be predicted based on cardiac remodelling during or after pregnancy and suggest potential areas of further research in the field.

## Introduction

Heart failure affects over 1 million people in the United Kingdom (1) and can be broadly subdivided into heart failure with reduced ejection fraction (HFrEF) and heart failure with preserved ejection fraction (HFpEF). The lifetime risk of developing any type of heart failure is 27.4% in men, and 23.8% in women (2). When analysed by subtype across the lifespan, the lifetime risk of developing HFrEF is greater in males, whereas the lifetime risk of developing HFpEF in similar between the genders (2). In terms of prevalence however, HFpEF disproportionately affects women (3), partly explained by a longer life expectancy in females, but also due to a difference in the pathophysiology and risk factors for heart failure between males and females.

There have been several reviews (4–7) in the last decade highlighting that there are gender differences in the pathophysiology and clinical manifestation of heart failure, but despite this little progress has been made in gaining a deeper understanding and management of the condition. Women diagnosed with heart failure (of any type), are more symptomatic and have a worse quality of life when compared to men diagnosed with heart failure. Yet, it takes longer for a woman to receive a diagnosis of heart failure (8). Women are poorly represented in heart failure trials, with no female-only heart failure trials, and the guidelines for treatment for heart failure are the same across both genders.

### Phenotypes of HFrEF and HFpEF

Many research groups have worked to define different phenotypes within heart failure, including using machine learning methods. Phenotyping heart failure is important as therapeutic approaches may vary based on the phenotype.

The Heart Failure Association (HFA) produced a consensus document in 2021, outlining 11 different potential phenogroups of patients with heart failure and reduced ejection fraction (9). These were based on heart rate and blood pressure, the presence of atrial fibrillation (AF) or sinus rhythm, the presence or absence of chronic kidney disease, the presence of hyperkalaemia, and the hypertensive patient. When applied in a real-world cohort, Radhoe et al. found that only 37% of patients with heart failure could be categorised into one of the patient profiles suggested by the HFA (10). 65% of the patients in Radhoe's cohort were male, and this male:female ratio was largely distributed evenly across most profiles, apart from HFA profile 11 (heart rate 60–70 bpm, blood pressure >90/60 mmHg, no AF, potassium >5.5 mmol/L) where 77% of the population were male. However, the numbers were small in this group (17 in total), and thus a repeat study in a female population would derive useful information regarding whether there is a particular profile more common in females. It must be noted however than over 60% of the population could not be categorised into one of the phenogroups and therefore Radhoe's results suggest a much wider and significant heterogeneity in the heart failure population.

In terms of HFpEF, between two and six phenogroups have been identified, mainly based on clinical and biochemical data and some echocardiographic input variables (11). No studies have been performed using magnetic resonance imaging (MRI) data variables, and a very limited number have used exercise data. Rucker and Joseph proposed three broad phenotypes of patients with heart failure and preserved ejection fraction (12). The first were young, with relatively few comorbidities but were obese and had low B-type natriuretic peptide (BNP) levels, the second were obese with multiple cardiometabolic coexistent pathologies, and the third were old, had multiple comorbidities and particularly high rates of AF and chronic obstructive pulmonary disease. Other studies have found similar phenotypes. For example, Uijl et al, identified 5 distinct clusters of patients with HFpEF, but in contrast to studies phenotyping HFrEF, they found a cluster more specific to women (13). Patients in cluster 5 (with a 100% presence of hypertension, 83% of atrial fibrillation, and 66% of diabetes) were much more likely to be an older (median age 82) and female (68% in the SwedeHF cohort and 80% in the CHECK-HF cohort). The baseline characteristics of the SwedeHF cohort included a median age of 80 years, with 52.4% females, and in the CHECK-HF cohort the median age was 77, with 54.5% females. Similarly, Pierre-Jean et al. found a cluster more specific to women, where the patient is more likely to be an older female, with fewer significant comorbidities and less advanced heart disease (14).

Importantly, no group has so far found a phenotype of heart failure related to a woman's pregnancy history. This is likely because details of pregnancy history have not been entered as a clinical data variable.

## Gender specific pathophysiology of heart failure

Conventional risk factors for heart failure include increasing age, hypertension, coronary artery disease, myocardial infarction, obesity, atrial fibrillation, and renal disease. These risk factors apply to both men and women. However, there are some risk factors for heart failure specific to women, which may help to explain the disproportionate amount of HFpEF found in women.

### Reproduction and pregnancy related risk factors

In a woman's life course, there is an increased risk of heart failure in her reproductive years and usually this associates with pregnancy. Acutely this can be a peripartum cardiomyopathy or pregnancy hypertension related heart failure, which can present as both heart failure with reduced or preserved ejection fraction (15). Pregnancy can also unmask a dilated cardiomyopathy (16).

### Normal cardiac remodelling in pregnancy

During pregnancy, there is a significant shift in maternal haemodynamics, with a decrease in peripheral vascular resistance, a consequent increase in renin-angiotensin-aldosterone system activity and a large increase in circulating plasma volume (17). This is accompanied by normal, physiological cardiac adaptation resulting in an increased left ventricular end-diastolic diameter, increased left ventricular wall thickness, increased left ventricular mass, increased left atrial size (18) and a small reduction in left ventricular diastolic function. Left ventricular systolic function remains preserved. These changes are thought to resolve between one- and six-months following pregnancy, with cardiovascular adaptations returning to a pre-pregnancy state, in a process known as reverse remodelling (19).

### Altered cardiac remodelling in pregnancy complicated by disease

In hypertensive pregnancy including pre-eclampsia, the aforementioned changes seen in a normotensive pregnancy are altered and exaggerated due to the increased pressure load. Whilst there is an increase in left ventricular end-diastolic diameter, there is an even greater increase in left ventricular wall thickness and mass relative to a normotensive pregnancy. Additionally, the pattern of increase in wall thickness is altered resulting in a disproportional increase in left ventricular mass and hypertrophy known as concentric remodelling (17, 18). The deterioration in left ventricular diastolic function is greater, and the changes persist on average for much longer than in women with normotensive pregnancy (20, 21). Melchiorre et al. showed that at a one-year follow up evaluation, asymptomatic left ventricular hypertrophy and dysfunction was significantly higher in women with pre-eclampsia compared with matched controls (21). However, there are some women who have a hypertensive pregnancy who do exhibit normalisation of their cardiac size and function postpartum, and some who do not (22), and understanding which factors help promote reverse remodelling is an important area for further research. Kitt et al. showed that by improving blood pressure control for an average of 40 days postpartum, in those affected by hypertensive pregnancy, there was beneficial cardiac remodelling at 6–9 month follow up, thus demonstrating an early intervention can result in more favourable remodelling outcomes (23).

Rarely, hypertension in pregnancy can lead to acute hypertensive heart failure characterised by rapid onset of pulmonary oedema. In such cases, left ventricular diastolic dysfunction can progress to systolic dysfunction and dilatation as the ventricle struggles to maintain cardiac output due to a mismatch in ventricular-vascular coupling (24).

Women who suffer from gestational diabetes also have increased left ventricular relative wall thickness, increased left ventricular mass indexed for body surface area, and reduced left ventricular global longitudinal strain compared to women without gestational diabetes (25, 26). Aguilera et al. showed that these changes were slower to recover in the women with gestational diabetes, with a lower degree of improvement in left ventricular diastolic indices and systolic strain at the 6 month follow up (26).

The cardiac changes in peri-partum cardiomyopathy are different to gestational hypertension and gestational diabetes, in that it is defined as left ventricular systolic dysfunction with an ejection fraction less than 45%, in the absence of any other cause of heart failure (27). The impaired ejection fraction may also be accompanied by left ventricular dilatation. The causes of peri-partum cardiomyopathy are incompletely understood but risk factors include black ancestry, pre-eclampsia, advanced maternal age, multiple gestation pregnancy and presence of pregnancy-related diabetes (28). It is now thought that most women with peri-partum cardiomyopathy have an underlying genetic predisposition and that vasculo-hormonal changes towards the end of pregnancy can trigger the condition in these susceptible individuals. It has been suggested that 50%–80% of women with peri-partum cardiomyopathy recover within 6 months, with recovery measured as improvement in left ventricular (LV) systolic function to an ejection fraction >50% (29).

### Long-term risk of heart failure following pregnancy

We are now increasingly understanding that the augmented risk of heart failure in pregnancy and postpartum is not limited to that period. Multiple epidemiological studies now show that the incidence of heart failure is higher in women with previous hypertensive disorders of pregnancy, even if they did not have acute heart failure at the time of pregnancy. Honigberg et al. followed up over 500,000 women in a Norwegian registry who had had pregnancies between 1980 and 2004 over a 30-year period (30). The group with a hypertensive disorder of pregnancy had lower rates of heart failure-free survival. Williams et al. studied hospitalisations for heart failure, in particular hospitalisations for HFpEF and found that re-admissions with HFpEF were twice as likely in a group of women with previous hypertensive pregnancy, with median time of onset being only 32.2 months post-delivery (31). Leon et al. followed up a UK cohort over 20 years and reported that compared to women without hypertensive pregnancy, women who had at least 1 pregnancy affected by pre-eclampsia had a hazard ratio of 2.13 for heart failure (32). In this cohort, 65% of all cardiovascular disorders had occurred in women aged below 40, in keeping with Williams' findings that events were occurring at a very young age. Women admitted with pregnancy related HFpEF were more likely to be of Black ethnicity, as well as older, and poorer (33), suggesting socioeconomic risk factors may play a role in addition to biological risk factors. Studies using imaging such as echocardiography to follow up women post hypertensive pregnancy have also shown changes more consistent with HFpEF even if a woman is currently asymptomatic. Boardman et al. showed that women 5–10 years post hypertensive pregnancy had increased left ventricular mass, increased left atrial volume, and reduced functional capillary density, compared to women who had normotensive pregnancy (34).

The risk of heart failure following peripartum cardiomyopathy is increased even despite initial LV systolic function recovery. Jackson et al. showed 13% of women had a subsequent deterioration in LV function occurring at a median of 2.9 years after recovery (35). Another study using cardiac magnetic resonance imaging to follow up women up to 7 years after peripartum cardiomyopathy, found subtle diastolic dysfunction, even though systolic function had recovered (36). Focal myocardial fibrosis was uncommon.

### Non-pregnancy related risk factors

Non-pregnancy related risk factors for heart failure in women include chemotherapy, or anti-cancer agents for female specific cancers e.g., breast cancer. A follow up of over 8,000 patients with breast cancer showed an almost three times (HR 2.71, CI 1.70–4.33) increased risk of heart failure in the first year of diagnosis compared to a matched cohort of women without breast cancer (37). An increased risk of heart failure (HR 1.28, CI 1.03–1.59) was still evident 10 years post-diagnosis. Patients receiving anthracyclines and trastuzumab were at the highest risk of developing heart failure (37).

Takotsubo cardiomyopathy is an acute type of heart failure that is overwhelmingly higher in females (female:male ratio almost 9:1) (38). It is thought to be triggered by emotional stress with the resulting surge in circulating catecholamines having a direct effect on the myocardium resulting in temporary stunning and reduced systolic function (39). The risk of recurrent Takotsubo cardiomyopathy is increased after the first episode.

## Discussion

### Why do women suffer disproportionately from HFpEF?

HFpEF was previously thought to develop due to left ventricular remodelling in response to increased afterload, however, research work in the last couple of decades has led to a shift in belief that HFpEF develops from a “systemic proinflammatory state” associated with microvascular disease, endothelial dysfunction, oxidative stress, myocardial remodelling, and fibrosis (40). Such systemic inflammation is seen in pre-eclampsia thus providing a plausible pathophysiological explanation for the association between pre-eclampsia and HFpEF. It is possible that other conditions associated with HFpEF such as diabetes and obesity, which are also thought to exhibit such low-grade systemic inflammation and endothelial dysfunction, affect men and women differently (41). Women could be prone to a pro-inflammatory state in these conditions than men, which might contribute to the increased prevalence of HFpEF in women. Indeed, many inflammatory conditions such as systemic lupus erythematosus or rheumatoid arthritis also have a female predominance.

Honigberg et al. explored the increased risk of HFpEF following hypertensive disorders of pregnancy by following up women included in the UK Biobank study (42). He found that the incidence of coronary artery disease, heart failure and even valvular disorders were higher in the hypertensive pregnancy cohort. Causal mediation analysis to investigate the proportional contribution of different factors such as hypertension or hypercholesterolaemia to the risk of development of these cardiovascular disorders revealed that 49% of a hypertensive pregnancy's association with heart failure is mediated by chronic hypertension. No significant mediation effect was found for hypercholesterolaemia and diabetes. Thus, some of the excess risk of long-term heart failure after pregnancy could be explained by the increased risk of later life hypertension, but >50% appears to be due to the hypertensive pregnancy and the cardiac remodelling that occurs during and after the pregnancy itself. This was corroborated by Boardman et al. who also showed that the adverse remodelling seen in the group of women with hypertensive pregnancy up to a decade postpartum was independent of office and ambulatory blood pressure (34).

### Is there a specific hypertensive pregnancy related HFpEF phenotype?

To investigate whether a hypertensive pregnancy-associated HFpEF phenotype exists, long-term follow up studies of women who have had a hypertensive pregnancy are needed. Serial cardiac imaging e.g., echocardiography, MRI or computed tomography (CT) pre-pregnancy, peripartum, and post-pregnancy would be invaluable in our understanding of the evolution of cardiac changes following hypertensive pregnancy compared to normotensive pregnancy and whether there is any sub-clinical evidence for early emergence of heart failure during or after hypertensive pregnancy. Further studies using causal mediation analysis are also required to investigate how hypertensive pregnancy is associated with specific imaging biomarkers of HFpEF, and whether the increased risk of HFpEF following hypertensive pregnancy is explained by later life hypertension alone or due to the multitude of other changes that occur during pre-eclampsia alongside raised blood pressure.

### Can we predict which women might develop cardiac problems later in life based on the remodelling during pregnancy or events during pregnancy?

It can be hypothesised that the long-term risk of heart failure can be predicted by how a woman's cardiovascular system responds following pregnancy. Predictors could include either the severity or pattern of cardiac remodelling after pregnancy, or the persistence of the remodelling before normalising. Alternatively, there could be an effect of pregnancy on the myocardium causing adverse sub-clinical changes e.g., fibrosis, which decreases myocardial reserve. It is easy to understand these changes in a pregnancy complicated by hypertension or diabetes or acute heart failure where there is increased inflammation, oxidative stress, and microvascular dysfunction, but it may be that even within “normal” pregnancy, changes can occur within a spectrum. Those with changes closer to the “adverse remodelling” end of the spectrum may be at greater risk of developing long-term heart failure. Factors pushing cardiac remodelling within “normal” pregnancy to the adverse end of the spectrum may be a genetic predisposition, certain ethnicities, age, antenatal blood pressure or presence of co-morbidities. It may be that when these women develop more conventional risk factors for heart failure later in life, e.g., hypertension or myocardial infarction, their cardiovascular system having already suffered one “hit” from adverse remodelling during pregnancy, is then “less able” to cope with the stress of cardiovascular disease, rendering it more susceptible to developing heart failure earlier.

### Clinical perspectives

Whilst it is evident that there is an elevated risk of cardiovascular events after hypertensive disorder of pregnancy, we are still unable to accurately predict which women might be most affected. Although clinicians can identify those who might be at increased risk from their pre-pregnancy risk factors e.g., higher age, raised BMI, presence of metabolic dysfunction, smoking history etc, there is no current way of using cardiac remodelling during or after pregnancy to assign future cardiovascular risk more accurately.

Research into this area is active, for example, Giorgione et al. developed a model based on maternal age, BMI, antenatal blood pressure and peripartum echocardiography which helped predict women at greatest risk of persisting hypertension at three months post-hypertensive pregnancy (43). Our research group is carrying out longitudinal multi-modality imaging studies of women post-hypertensive pregnancy aiming to identify specific remodelling patterns and early disease biomarkers which can be used in risk prediction models, and to design intervention trials aimed at reducing future cardiovascular risk. If it can be demonstrated for example, that presence of a certain feature, can predict early onset disease which could then be mitigated by an intervention, then this could help drive forward a guideline recommending cardiac imaging for all women during or post hypertensive pregnancy. Similarly, long term follow-up studies of women with other cardiovascular disease states during pregnancy e.g., peripartum cardiomyopathy are also needed, to help with accurate risk determination and generation of screening guidelines and intervention trials.
